# Transmission potential of Andes virus from human to human

**DOI:** 10.1016/j.nmni.2026.101768

**Published:** 2026-05-20

**Authors:** Hiroshi Nishiura, Yuri Amemiya

**Affiliations:** Graduate School of Medicine, Kyoto University, Yoshidakonoecho, Sakyoku, Kyoto, 6068501, Japan

**Keywords:** Hantavirus, Hantavirus pulmonary syndrome, Basic reproduction number, Transmissibility, Epidemiology

Dear editor,

An outbreak of Andes virus (ANDV), a type of hantavirus (a genus within the *Hantaviridae* family) known to cause hantavirus pulmonary syndrome, has been reported on a cruise ship that departed from Argentina in April 2026. While the natural reservoir is long-tailed pygmy rice rat which is unique to South America, only ANDV among hantaviruses has a capacity to involve a certain number of transmissions from human to human. Because the reservoir rat is very specific to the virus, importation of human cases in Europe or North America does not immediately imply the introduction of disease for an extended period of time including in animal species. While human-to-human transmission of ANDV is also subjectively regarded as infrequent (due to the absence of pandemic by now), the transmission potential from human to human is the key factor to judge the absence of continued local outbreak, and thus, it is vital to firmly understand the transmissibility. Here we aimed to estimate the basic reproduction number *R*_0_ of ANDV except for the still-developing ongoing outbreak on cruise ship.

We systematically collected documented human-to-human transmission events of ANDV (Table 1). In total, there were eight published studies from 1993 to 2019 [[1], [2], [3], [4], [5], [6], [7], [8]], including three from Chile and five reported from Argentina. There were 339 introductions to human population, involving a total of 448 human cases. Of 339 primary cases, 44 (13.0%) experienced at least one secondary transmission. Of these, 29 had only one secondary case, while 8, 4 and 1 introductions, respectively, resulted in a total of 3, 4 and 5 cases in each cluster. Other than these, two big clusters with 16 and 34 cases in total were reported in 1996-7 and 2018-9, respectively.

**Table:** Cluster size distribution of documented human infections of Andes virus, 1993-2019.

Country	Year	Ref	Total cluster size (persons)						
			1	2	3	4	5	16	34
Argentina	1993-2005	1	31	8	0	1	0	0	0
Chile	1995-2012	2	80	4	5	0	0	0	0
Argentina	1996-7	3	0	0	0	0	0	1	0
Chile	1997-8	4	22	1	0	1	1	0	0
Chile	2001-5	5	62	12	2	0	0	0	0
Argentina	2002	6	18	1	1	2	0	0	0
Argentina	2009-14	7	82	3	0	0	0	0	0
Argentina	2018-9	8	0	0	0	0	0	0	1
Total	1993-2019		295	29	8	4	1	1	1

Ref, references. Total cluster size included primary case, and thus, the category of 1 represents the absence of secondary transmission.

Using the total documented cluster size distribution, and employing a negative binomially distributed offspring distribution which is known to capture realistic skewed patterns of secondary transmission, *R*_0_ and dispersion parameter *k* of human-to-human ANDV transmission were estimated at 0.24 (95% confidence interval (CI): 0.18, 0.34) and 0.14 (95% CI: 0.08, 0.25), respectively.

As an alternative approach, it is known that the proportion of cases with environmental exposure is equal to 1- *R*_0_. In the observed data, out of 448 cases in total, there were 339 primary cases, and thus 75.7% of cases literally had an exposure to rodent or contaminated environment. Accordingly, *R*_0_ was estimated at 0.24 (95% CI: 0.20, 0.28).

In both analyses, *R*_0_ was estimated at 0.24 and was sufficiently below the value of one. Because sporadic cases are less likely ascertained than large clusters, and thus, it is likely that cluster size of one (i.e. no secondary case) is perhaps underestimated in its frequency, we may have overestimated *R*_0_. Thus, the actual transmissibility may be even smaller than what is presented here. Accordingly, assuming that the virus characteristics have not substantially changed from that causing past outbreaks in Table 1, we can objectively conclude that it is very unlikely that the outbreak that started from the cruise ship may not continue with sustained human-to-human transmission.

Apart from *R*_0_, it must be noted that the secondary transmission is over-dispersed (i.e. accompanied by a large variance) with *k* = 0.14. This implies that the heterogeneity is highly influential, sometimes allowing a cluster to involve more than 10 human cases. Optimal conditions that allow such super-spreading event must be clarified in a neat epidemiological and environmental investigations, examining whether a large-scale transmission can be attributed to characteristics of primary case, those of exposed susceptible individuals, virus characteristics or environmental settings. Identifying environmental conditions allowing transmission of aerosolized particles is a common scientific task shared with investigation of environmental exposure, including whether the large cluster on the cruise ship had something to do with environmental exposure with limited air exchange rate.

## CRediT authorship contribution statement

**Hiroshi Nishiura:** Conceptualization, Formal analysis, Investigation, Methodology, Validation, Writing – original draft, Writing – review & editing. **Yuri Amemiya:** Data curation, Investigation, Methodology, Writing – original draft, Writing – review & editing.

## Declaration of competing interest

The authors declare that they have no known competing financial interests or personal relationships that could have appeared to influence the work reported in this paper.
